# Everyday cognition scales are related to cognitive function in the early stage of probable Alzheimer’s disease and FDG-PET findings

**DOI:** 10.1038/s41598-017-01193-6

**Published:** 2017-05-11

**Authors:** Jung-Lung Hsu, Wen-Chuin Hsu, Chiung-Chih Chang, Kun-Ju Lin, Ing-Tsung Hsiao, Yen-Chun Fan, Chyi-Huey Bai

**Affiliations:** 1 Department of Neurology, Chang Gung Memorial Hospital Linkou Medical Center and College of Medicine, Chang-Gung University, Taoyuan, Taiwan; 2 Graduate Institute of Humanities in Medicine, Taipei Medical University, Taipei, Taiwan; 3 Brain and Consciousness Research Center, Taipei Medical University, Taipei, Taiwan; 4 Dementia Center and Section of Dementia, Department of Neurology, Chang Gung Memorial Hospital, Taoyuan, Taiwan; 5 Department of Neurology, Cognition and Aging Center, Kaohsiung Chang Gung Memorial Hospital, Chang Gung University College of Medicine, Kaohsiung, Taiwan; 6 Department of Nuclear Medicine and Center for Advanced Molecular Imaging and Translation, Linkou Chang Gung Memorial Hospital, Taoyuan, Taiwan; 7 Department of Medical Imaging and Radiological Sciences and Healthy Aging Research Center, Chang Gung University, Taoyuan, Taiwan; 8 School of Public Health, College of Public Health, Taipei Medical University, Taipei, Taiwan; 9 Department of Public Health, College of Medicine, Taipei Medical University, Taipei, Taiwan

## Abstract

We applied the Everyday Cognition (ECog) scale in normal aging adults and patients with Alzheimer’s disease (AD) to investigate associations between neuropsychological tests and neuroimaging markers. A total of 160 normal aging adults and 40 patients with the early stage of probable AD were included. Neuropsychological performance was assessed using the Consortium to Establish a Registry for Alzheimer’s Disease Neuropsychological Assessment Battery (CERAD-NAB). ^18^F-fluorodeoxyglucose positron emission tomography (FDG-PET) scans were used to measure AD-related hypometabolism. Nonparametric Spearman correlation analysis was used to study associations between ECog and z-transformed total CERAD-NAB scores in both groups. The results revealed a significant correlation between total ECog and CERAD-NAB scores (rho = −0.28, p < 0.01), and category verbal fluency test with the executive domain of the ECog scale (rho = −0.20, p < 0.01). The CERAD-NAB scores were also significantly correlated with AD-related hypometabolism (rho = −0.49, p < 0.01). The memory domain of the ECog scale was significantly correlated with FDG uptake in the angular gyrus and posterior cingulum gyrus (rho = −0.41 and −0.46, *P* < 0.01). In conclusion, both total and memory domain ECog scores were correlated with the neuropsychological tests and neuroimaging biomarkers.

## Introduction

Of the dementia syndromes, Alzheimer’s disease (AD) is the most common degenerative form. Its diagnosis is based on core clinical features and supported by neuroimaging studies or cerebrospinal fluid markers^1^. The most prominent cognitive deficit in early stage AD is episodic memory loss, which can be detected by performance-based neuropsychological tests^2, 3^. Functional impairments or a decline from previous levels in performance are also included in the core diagnostic criteria^1^, and can be assessed by informant-rated instruments such as the Clinical Dementia Rating (CDR) scale^4^, basic activities of daily living (ADLs) or “instrumental” activities of daily living (IADL)^5, 6^. The early detection of this functional decline has been shown to have prognostic value in predicting future disease progression or conversion to dementia^7, 8^. In addition, the functional decline in AD has been linked to objective biomarkers such as cortical gray matter loss^9^, small hippocampus volume^10^, and increased amyloid burden on positron emission tomography (PET) scans^11^. However, the relationship between functional activity and ^18^F-fluorodeoxyglucose positron emission tomography (FDG-PET) has yet to be fully elucidated. The Everyday Cognition (ECog) scale is a validated informant-rated questionnaire that includes one global factor and six domain-specific factors^12^. The psychometric properties in the ECog scale focus on mild problems in everyday function and cognition that may complement and expand those included in older informant-rated instruments.

The Consortium to Establish a Registry for Alzheimer’s Disease Neuropsychological Assessment Battery (CERAD-NAB) is a well-validated, widely used tool to study cognitive deficits in patients with dementia^13, 14^. It has been translated into several languages including German, Finnish, and simplified and traditional Chinese for use in Hong Kong, China and Taiwan^15–20^. The total score of the CERAD-NAB has been shown to have good accuracy in diagnosing the early stage of AD in several multi-center studies^21, 22^. Moreover, in a brain-behavior study, the CERAD-NAB was significantly associated with regional cerebral metabolism as measured by FDG-PET^23^. However, associations between the ECog scale and neuropsychological measurements have not been extensively studied. Previous studies have shown that the ECog scale is significantly correlated with global cognition and individual domains of cognitive tests^12, 24^. Furthermore, other functional tools such as the AD8 have been shown to be superior to performance-based cognitive measurements in identifying the underlying pathology in the early stage of AD^25^. Although one neuroimaging study demonstrated that episodic memory and hippocampal volume were associated with each domain of the ECog scale in patients with AD^24^, the association between ECog and neuronal metabolism measured using FDG-PET is unknown. According to Jack’s hypothetical model of dynamic biomarkers in AD, abnormalities in FDG-PET can be detected earlier than changes in volumetric magnetic resonance imaging (MRI)^26^.

In the current study, we investigated associations among the ECog scale, CERAD-NAB and FDG-PET scans in the early stage of probable AD. We hypothesized that the ECog score may be correlated with neuropsychological results and FDG-PET abnormalities. In addition, we compared the sensitivity and specificity between the ECog scale and CERAD-NAB in the diagnosis of AD.

## Results

### Sample characteristics and cognitive measurements

A total of 160 normally aging adults and 40 patients with the early stage of probable AD were included for analysis. There were no significant differences in mean age, gender and years of education between the two groups. The years of education ranged from 0 to over 20 (mean and standard deviation for the normally aging group = 8.7 (4.6) and AD group = 9.2 (4.6)), with 25% of the participants having less than 6 years of education. Table 1 shows the demographic characteristics of the participants. Overall, 47.5% of the informants were a spouse of the participant, 42.5% were an adult child, son-in-law/daughter-in-law of the participant, and 10% were reported as ‘other’. There were significant differences in mean MMSE scores (26.4 ± 3 vs. 22.4 ± 4, Z = −5.48, *P* < 0.01) and total CERAD-NAB scores (75.5 ± 9 vs. 62.6 ± 11, Z = −6.33 *P* < 0.01; Mann–Whitney U test) between the normally aging and probable AD groups. In the early stage of probable AD group, 38 patients had a CDR score of 0.5 and two had a CDR score of 1. In the normally aging group, the regression analysis of total CERAD-NAB score showed a significant association with age and years of education (*P* < 0.01), but not with gender (*P* = 0.58). The R-square of regression was 0.42 (*P* < 0.01).

**Table 1:** Demographic characteristics of the normally aging and the early stage of probable AD groups.

Group	Normal aging subjects (n = 160)	Early stage of probable AD (n = 40)	P value^a^
Age, years (mean ± SD)	70.4 ± 9.3	67.8 ± 8.0	0.12
Gender, n (%)			0.97
Male	61 (38.1)	16 (40)	
Female	99 (61.9)	24(60)	
Education, years (mean ± SD)	8.7 ± 4.6	9.2 ± 4.6	0.72
Total score of CERAD-NAB (mean ± SD)	75.5 ± 9.0	62.6 ± 11.5	<0.01
MMSE (mean ± SD)	26.4 ± 3.1	22.9 ± 4.2	<0.01
CDR, median (IQR)	0 (0–0)	0.5 (0.5–0.5)	<0.01
CDR-SB, median (IQR)	0 (0–0)	2.0 (1.1–2.5)	<0.01

Abbreviations: SD, standard deviation; AD, Alzheimer’s disease; MMSE, Mini Mental State Examination; CERAD-NAB, Consortium to Establish a Registry for Alzheimer’s Disease Neuropsychological Assessment Battery; CDR, Clinical Dementia Rating; CDR-SB, Clinical Dementia Rating-sum of box; IQR, interquartile ranges. ^a^Tested by the chi-square tests and the Mann-Whitney U test.

### Characteristics of the ECog scale and correlations with the CERAD-NAB test

**Figure 1:**
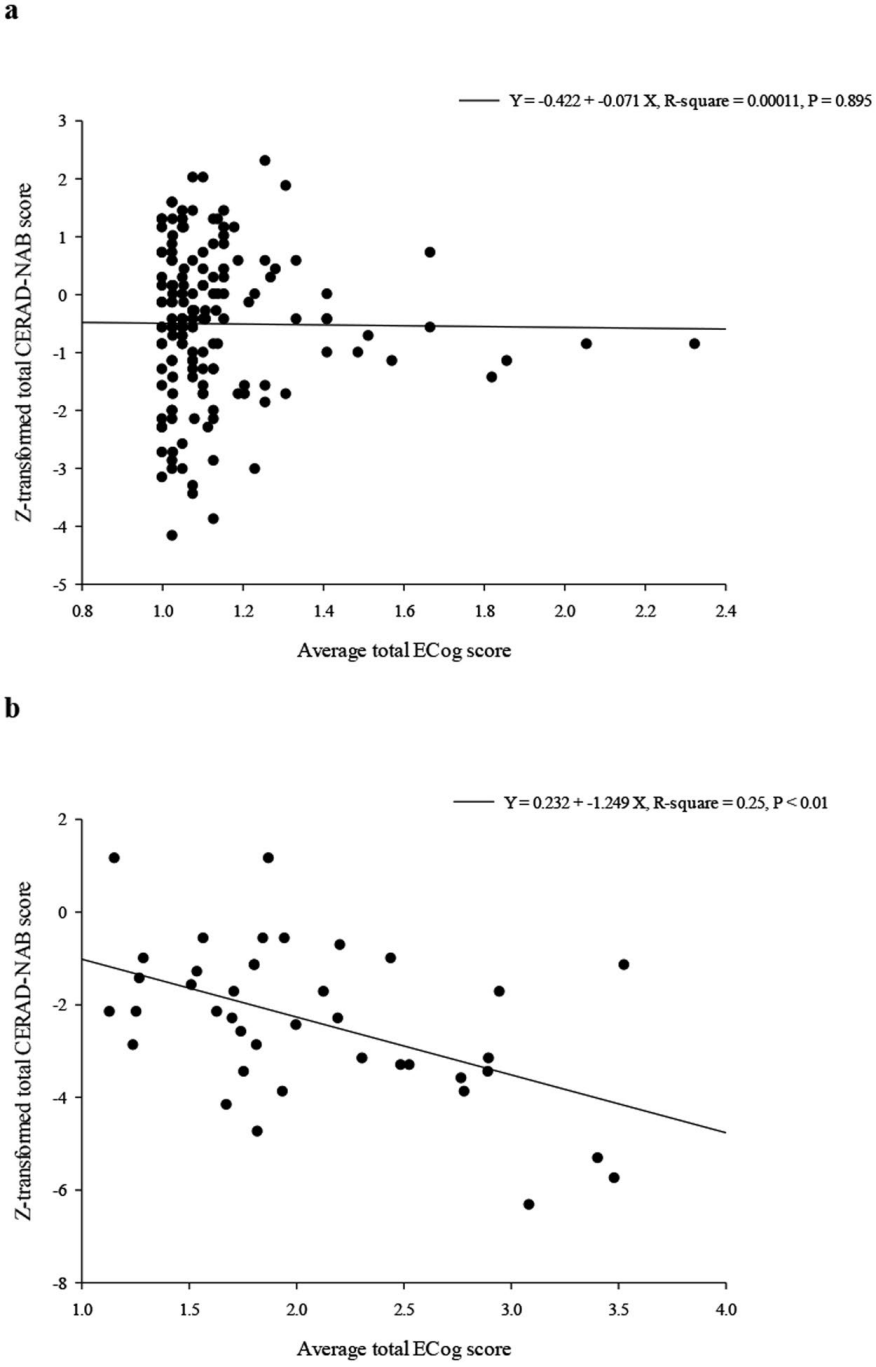
Scatter plot between average total ECog score and z-transformed CERAD-NAB score in (**a**) normally aging and (**b**) early stage of probable AD groups.

**Figure 2:**
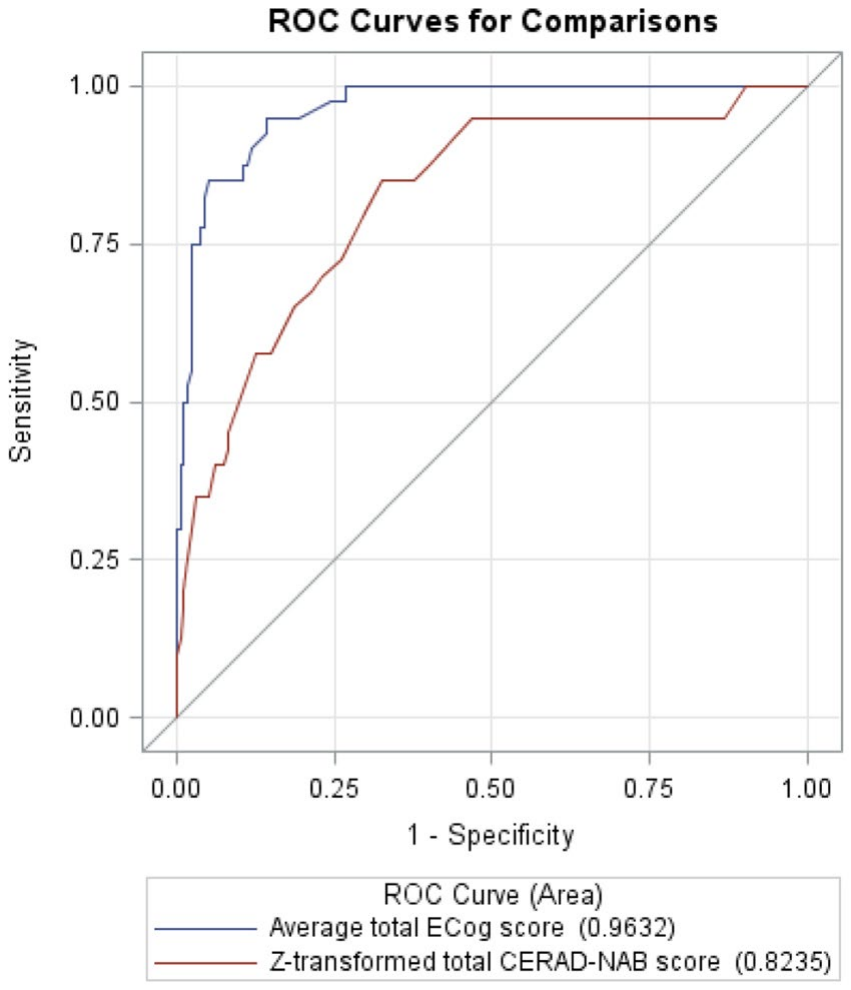
ROC curves of z-transformed total CERAD-NAB score and average total ECog score. The ROC curves were tested for the ability to discriminate between normally aging adults and patients with early stage probable AD. The AUC associated with the average total ECog scores was significantly higher than that associated with the z-transformed total CERAD-NAB score (AUC = 0.96, 95% CI = 0.94–0.99, sensitivity = 0.95, specificity = 0.86, cutoff point = 1.23; AUC = 0.82, 95% CI = 0.75–0.90, sensitivity = 0.85, specificity = 0.68, cutoff point = −1.01, respectively; *P* < 0.01).

The total ECog scores and average ECog scores were significantly lower in the normally aging group than in the early stage of probable AD group (total ECog score: 43.5 ± 7 vs. 76.9 ± 24, Z = −8.60; average ECog score: 1.1 ± 0.2 vs. 2.1 ± 0.7, Z = −9.07; Mann–Whitney U test all *P* < 0.01). The mean ECog scores in each domain were all significantly lower in the normally aging group than in the early stage of probable AD group (Mann–Whitney U test all *P* < 0.01) (Table 2). We used regression analysis to study the association between total ECog scores and age, gender, and years of education in the patients with probable AD and controls. The results showed that total ECog scores were not significantly associated with age, gender or years of education (*P* = 0.18 for age, *P* = 0.89 for gender and *P* = 0.70 for years of education). Pairwise correlation analysis revealed a significant correlation between the total ECog scores and total CERAD-NAB scores in all participants (Spearman correlation coefficient = −0.28, *P* < 0.01). The word-list learning test with delayed recall and recognition from the CERAD-NAB test showed a significant correlation with all domains of the ECog scale (Spearman correlation coefficient from −0.25 to −0.44, all *P* < 0.01). The category verbal fluency test also showed a significant correlation with planning and organization domains of the ECog scale (Spearman correlation coefficient = −0.20 and −0.21 respectively, both *P* < 0.01; Table 3). Moreover, a scatter plot demonstrated that the average total ECog score was significantly correlated with the z-transformed CERAD-NAB score in the early stage of probable AD group (*P* < 0.01) (Fig. 1). We then compared the discriminative abilities of the average total ECog score and z-transformed total CERAD-NAB score between the two groups using the Wald test. The AUC associated with the average total score of the ECog scale was significantly higher than that associated with the z-transformed total CERAD-NAB score (AUC = 0.96, 95% CI = 0.94–0.99, sensitivity = 0.95, specificity = 0.86; AUC = 0.82, 95% CI = 0.75–0.90, sensitivity = 0.85, specificity = 0.68, respectively; *P* < 0.01; Fig. 2).

**Table 2:** Total ECog scores and average score of six domains in the two groups.

Group	Normally aging (n = 160)	Early stage of probable AD (n = 40)	P value^a^
Total ECog score	43.6 ± 7.0	76.9 ± 23.5	<0.01
Average total ECog score	1.1 ± 0.2	2.1 ± 0.7	<0.01
Average memory domain	1.3 ± 0.4	2.8 ± 0.8	<0.01
Average language domain	1.1 ± 0.2	1.7 ± 0.7	<0.01
Average visual-spatial and perceptual abilities	1.1 ± 0.2	1.8 ± 0.7	<0.01
Average planning domain	1.0 ± 0.2	1.8 ± 0.7	<0.01
Average organization domain	1.0 ± 0.1	1.9 ± 0.8	<0.01
Average divided attention domain	1.3 ± 0.4	2.4 ± 1.0	<0.01

Abbreviations: AD, Alzheimer’s disease; ECog, Everyday Cognition. Data was expressed as mean ± SD. ^a^Tested by the Mann-Whitney U test.

**Table 3:** Pairwise correlation between four domains of CERAD-NAB score and six domains of the ECog score in all participants.

	Word-list learning test with delayed recall and recognition	Modified Boston Naming Test	Visual construction Test	Category verbal fluency Test	Total CERAD scores
Average memory domain	−0.33*	−0.06	−0.05	−0.14	−0.31*
Average language domain	−0.34*	−0.10	−0.06	−0.16	−0.31*
Average visual-spatial and perceptual abilities domain	−0.30*	−0.07	0.03	−0.16	−0.28*
Average planning domain	−0.38*	−0.05	0.013	−0.20*	−0.36*
Average organization domain	−0.44*	−0.12	−0.02	−0.21*	−0.41*
Average divided attention domain	−0.25*	−0.04	−0.06	−0.13	−0.25*
Total ECog score	−0.30*	−0.10	−0.02	−0.10	−0.28*

Abbreviations: ECog, Everyday Cognition; CERAD-NAB, Consortium to Establish a Registry for Alzheimer’s Disease Neuropsychological Assessment Battery Tested by spearman’s rank correlation. *P < 0.01.

### AD-related hypometabolism was correlated with ECog scale and CERAD-NAB test results in the early stage of probable AD group

To further assess the relationship between AD-related hypometabolism derived from FDG-PET scans and clinical measurements, we performed nonparametric pairwise correlation analysis. The z-transformed total CERAD-NAB scores were significantly negatively correlated with the AD t-sum scores (Spearman correlation coefficient = −0.49, *P* < 0.01). The average total score of the ECog scale was not significantly correlated with the AD t-sum scores. Only the average score of the memory domain of the ECog scale demonstrated a trend of correlation with the AD t-sum scores (Spearman correlation coefficient = 0.36, *P* = 0.02; Table 4). There were no significant correlations between other ECog domains and AD t-sum scores.

**Table 4:** Pairwise correlation analysis between average memory domain of ECog, average total ECog, z-transformed CERAD-NAB score and PALZ scores in patients with early stage of probable AD.

	Average Memory domain	Average total ECog score	z-transformed total CERAD-NAB score	AD t-sum score
Average Memory domain	1.00	0.86*	−0.48*	0.36
Average total ECog score	0.86*	1.00	−0.40*	0.22
z-transformed total CERAD-NAB score	−0.48*	−0.40*	1.00	−0.49*
AD t-sum score	0.36	0.22	−0.49*	1.00

Abbreviations: ECog, Everyday Cognition; CERAD-NAB, Consortium to Establish a Registry for Alzheimer’s Disease Neuropsychological Assessment Battery; AD, Alzheimer’s disease. Tested by spearman’s rank correlation. *P < 0.01.

In regional analysis of FDG-PET scans, both the memory domain of the CERAD-NAB score and the z-transformed total CERAD-NAB scores were significantly correlated with the SUVr from the angular gyrus and posterior cingulum gyrus (Spearman correlation coefficients from 0.38–0.65, all *P* < 0.01). In addition, the executive domain of the CERAD-NAB score was significantly correlated with the SUVr of the posterior cingulum gyrus (Spearman correlation coefficient = 0.38, *P* < 0.01). The memory domain of the ECog scale also demonstrated a significant inverse association with the SUVr of the angular gyrus and posterior cingulum gyrus (Spearman correlation coefficients = −0.41 and −0.46 respectively, both *P* < 0.01; Table 5).

**Table 5:** Pairwise correlation analysis between regional SUVr, different cognitive domains of CERAD-NAB and memory domain of ECog scale in patients with early stage of probable AD.

	Executive domain of CERAD-NAB	Language domain of CERAD-NAB	Memory domain of CERAD-NAB	Visuospatial domain of CERAD-NAB	z-transformed total CERAD-NAB	Average memory domain of ECog scale
Middle frontal gyrus	−0.03	−0.47	−0.12	0.02	−0.12	0.07
Angular gyrus	0.28	0.05	0.65*	0.12	0.58*	−0.41*
Calcarine gyrus	0.26	0.25	0.13	0.16	0.22	−0.10
Middle temporal gyrus	0.16	0.20	0.21	−0.05	0.20	−0.16
Posterior cingulum gyrus	0.38*	0.03	0.39*	0.16	0.38*	−0.46*

Abbreviations: SUVr, Standard Uptake Value ratio; ECog, Everyday Cognition; CERAD-NAB, Consortium to Establish a Registry for Alzheimer’s Disease Neuropsychological Assessment Battery; AD, Alzheimer’s disease. Tested by spearman’s rank correlation. *P < 0.01.

## Discussion

In this study, we investigated everyday cognitive function using the ECog scale and compared the scores with those of the CERAD-NAB test. The results revealed that the total and average ECog scores were significantly associated with the neuropsychological test scores. The total ECog scores and all average domain-specific scores were significantly correlated with the CERAD-NAB memory subtest and total CERAD-NAB scores. The strength of the correlation was weak to moderate. In addition, two average scores of the executive domain (planning and organization domain) in the ECog scale were significantly correlated with executive CERAD-NAB subtest score. Using a functional tool, we demonstrated that the ECog scale could differentiate patients with the early stage of probable AD from normally aging adults. In the AUC analysis conducted to discriminate both groups, the average total score of the ECog scale was associated with a significantly higher AUC value compared with the z-transformed total CERAD-NAB score. Furthermore, the memory domain of the ECog scale and CERAD-NAB score were both significantly associated with the regional SUVr in the angular gyrus and posterior cingulum gyrus, suggesting their diagnostic potential for the early stage of AD.

The ECog scale is a useful tool for measuring general and domain-specific everyday functions in elderly people, and in patients with mild cognitive impairment (MCI) and dementia. There were significant differences in total score and the six average domain-specific scores between the normally aging and early stage of probable AD groups, which is consistent with the findings of a previous study^12^. Moreover, this functional score was significantly negatively correlated with the CERAD-NAB score, suggesting that functional impairment is related to cognitive dysfunction^12^. In a regression study, the average total ECog score was significantly negatively correlated with z-transformed total CERAD-NAB scores in patients with the early stage of AD (*P* < 0.01) but not in normally aging adults, which may be due to the flooring effect of the ECog scale. In the current study, the average domain-specific scores were significantly correlated with memory subtest scores of the CERAD-NAB test. The results indicated that memory performance may be associated with memory and other cognitive domains of the ECog scale. Executive function as assessed using the category verbal fluency test was also significantly correlation with the executive domain of the ECog scale, which is compatible with the dysfunction of executive ability in the early stage of AD^27, 28^. However, the cognitive deficits revealed in the CERAD-NAB test but not in the ECog scale were associated with AD t-sum scores, as observed in FDG-PET, demonstrating that cognitive dysfunction may not be completely equal to functional impairment. In confirmatory factor analysis of the ECog scale, one global factor and six domain-specific factors have been reported^12^. Other factors such as neuropsychiatric symptoms (e.g., depression and apathy) have also been reported to independently contribute to the ECog score^29^. These results support that functional impairment and cognitive dysfunction share a common factor but are also independent.

The discriminative ability of the average total ECog score between the two groups was associated with a significantly higher AUC value compared with the z-transformed total CERAD-NAB score. Most cognitive tests are performance-based measurement tools that require a group of age- and education-matched normal participants for comparison to demonstrate deficits. However, the ECog scale is weakly correlated only with age and years of education, which could be beneficial when studying a group with a wide range of years of education as in the current study^12^. In addition, an informant-based assessment tool provides an opportunity for clinicians to assess changes in a patient’s functioning level and determine the interference in their daily tasks, whereas a neuropsychological battery is a performance-based measurement tool that requires a group of age- and education-matched normal participants for comparison to demonstrate the deficits. Theoretically, compared with a measurement tool for inter-participant comparisons, a tool for intra-participant comparisons could be more sensitive in detecting the subtle changes in the early stages of AD^25, 30^. Our results demonstrated that the memory domain score of the ECog scale was associated with regional FDG uptake in the bilateral angular gyrus and posterior cingulum gyrus. These findings suggest that the ECog scale may reflect cortical regional metabolism status. Furthermore, the ECog scale includes numerous everyday cognitive domains other than memory function, such as language, visual–spatial, and executive function. Various dementia syndromes such as behavioral variants of frontotemporal dementia, posterior cortical atrophy, and primary progressive aphasia may initially present as distinct everyday cognitive deficits rather than memory problems, which could possibly be identified using the ECog scale^31^. The ECog scale may therefore potentially help clinicians to more efficiently differentiate among degenerative dementia syndromes.

In a neuroimaging study, Farias *et al*. reported that the hippocampal volume and total brain volume were associated with most ECog domains, whereas the frontal lobe volume was independently associated with two everyday executive domains in normal participants and in patients with MCI and AD^24^. According to a review of the relevant literature, no previous study has investigated associations between the ECog scale and FDG-PET scans in relation to dementia. We found that the memory domains of the ECog were significantly correlated with FDG uptake in the angular gyrus and posterior cingulum gyrus in the patients with the early stage of probable AD (*P* < 0.01), whereas other non-memory domains of the ECog scale were neither correlated with AD t-sum scores nor regional SUVr from FDG-PET scans. The reason for this may be because the disease severity of our patients with the early stage of probable AD was very mild (mean CDR-SB = 2), and because most abnormal hypometabolism regions in the mild stage of AD are related to the memory domain. Of all CERAD-NAB test items, the memory-related domain contributed 50% (e.g., 3 word-list learning tests with delayed recall and recognition), whereas of all ECog scale items, the memory domain contributed only 23.6% (e.g., memory domain items: 8, total ECog items: 39). Another possibility is that the ECog scale is composed of six cognitive domains, and that the association between average total ECog scores and AD t-sum scores may decrease in patients with the early stage of probable AD. On the basis of this finding and because all six ECog domains exhibited a significant difference between the normally aging adults and early stage of probable AD groups, we suggest that in patients with the early stage of probable AD, the functional impairment measurements from the ECog scale can provide complementary information in addition to that from the performance-based cognitive tests.

There are several limitations to the present study. First, due to the low number of patients with the early stage of probable AD and a lack of patients with MCI, we could not generalize our results to clinical practice. The low number of normally aging adults may also have affected the z-transformed total CERAD-NAB score. In addition, we did not perform further studies in our normally aging adult group which may have included patients with MCI or even the early stage of AD. These factors may have decreased the correlation between ECog scores and CERAD-NAB scores. We used the CERAD-NAB test scores and MRI scans in the diagnostic process, however not the FDG-PET scans or ECog scores. According to the hypothetical model of dynamic biomarkers in AD, this process may have limited the enrollment of patients with probable AD at an earlier stage, and may have led to more significant differences in ECog scores between the normally aging adults and patients. It would be interesting to investigate whether both ECog scale and FDG-PET scans can improve the diagnostic repertoire in future studies. Second, the diagnosis of probable AD in this study was based on clinical criteria rather than neuropathological evidence. The FDG-PET scans only represented the neuronal injury markers in the diagnostic criteria. As the number of patients with amyloid biomarkers in this study was insufficient to perform subgroup analysis, further studies are needed to investigate the relationships between ECog scores and FDG-PET scans in patients with AD who are positive for amyloid markers. Third, we did not apply the ECog scale using self-rated participant tests and did not assess the mood characteristics or caregiver burden of the informants. A recent study revealed that self-reported complaints as measured by self-rated ECog scale were not correlated with cognitive dysfunction but with depressive symptoms, and that this may confound the misclassification of MCI^32^. We will consider this aspect in our next MCI study. Caregiver distress has been shown to affect the patient’s behavior rating in previous studies, which may in turn affect the ECog scores^12, 33^. Other limitation including incorporation bias in the ECog scale and CERAD-NAB test may also have confounded our findings. The application of these approaches in a large cohort of participants with MCI in the future is warranted.

In summary, the present study is the first to provide the results of applying the ECog scale to normally aging adults and patients with the early stage of probable AD. The ECog scores exhibited significant correlations with standard neuropsychological tests and could also discriminate between both groups. The memory domain of this functional ability scale and CERAD-NAB scores were associated with AD-related hypometabolism derived from FDG-PET scans.

## Methods

### Participants

We recruited 160 normally aging adults aged 50 years or older from several senior citizen community centers whose first language was Chinese or Taiwanese. Registered nurses, social workers, and occupational therapists reviewed the recent medical records of all participants. No memory or other cognitive complaints were confirmed in the participants before they were enrolled into the study, and they all underwent detailed physical and neurological examinations. Those with a history of major neurological, psychiatric, or severe cardiovascular diseases were excluded.

In addition, we recruited 40 patients aged older than 50 years from the outpatient clinics of our hospital as the disease group. In this study, the early stage of probable AD was diagnosed according to the criteria of the National Institute on Aging-Alzheimer’s Association^1^. Cognitive dysfunction was assessed using the CERAD-NAB test, and brain MRI scans were used to exclude the possibility of structural brain lesions such as hydrocephalus or brain tumors. The severity of dementia measured by the Clinical Dementia Rating (CDR) scale score was limited to 0.5 to 1^4^. All procedures were approved by the Institutional Review Board (IRB) of Chang Gung Memorial Hospital, and all participants signed informed consent to participate in the study (IRB number:103-1994B). The study was performed in accordance with the approved guidelines and regulations.

### ECog assessment and neuropsychological battery

The informants of the normally aging adults and the patients with the early stage of probable AD completed the ECog scale. The ECog scale is comprised of 39 items covering six domains (Everyday Memory, Language, Visual-spatial and Perceptual Abilities, Planning, Organization, and Divided Attention), and is scored based on a four-point scale: 1 = better or no change compared to 10 years earlier, 2 = questionable/occasionally worse, 3 = consistently a little worse, 4 = consistently much worse. An “I don’t know” response option is also included. The total score was calculated as the sum of all 39 items, and the average score was derived from the mean average of all responses. If the response was “I don’t know”, then the item was not included in the calculation. The response rate for each item was calculated as the total number of responses from 1–4 (not including “I don’t know” responses) divided by the total number of participants. The translation and adaptation of the ECog scale involved a bilingual member of the medical staff translating the original ECog version to the Chinese version, followed by a neurologist with a subspecialty in dementia reviewing the translation and applying the translated version to a small group of early stage dementia caregivers.

The neuropsychological battery included the CERAD-NAB, Mini Mental State Examination (MMSE) and CDR scales, which were evaluated by a single rater for all participants^34^. The CERAD-NAB is comprised of five subtests derived from previously established cognitive tests: an executive domain of the category verbal fluency test^35^, a language domain of the Modified Boston Naming Test (BNT)^36^, a memory domain of the Word-List-Learning test with delayed recall and recognition^37^, and a visuospatial domain of the visual construction test^38^. The total score of the CERAD-NAB was calculated by summing the six subtest scores (category verbal fluency [maximum score = 24], modified BNT [maximum score = 15], word-list learning [maximum score = 30] with delayed recall [maximum score = 10] and recognition [maximum score = 10] and visual construction test [maximum score = 11]). The CDR sum of box (CDR-SB) was calculated as the sum of all six items in the CDR scale.

### FDG-PET study in the patients with the early stage of probable AD

All of the patients underwent brain MRI and FDG-PET scans on an integrated PET-MR system (Siemens Biograph mMR scanner), which could simultaneously acquire PET and MRI data using a vendor-supplied 12-channel phase-array head coil according to standard procedures. In brief, the participants were required to fast for 4 h before imaging. FDG data were collected 30 min after the injection of a dose of 185 MBq FDG. This 30-min PET data reflected the distribution of FDG tracer after homogeneous and complete distribution in the brain. A high-resolution magnetization-prepared rapid acquisition gradient-echo T1-weighted image (voxel size 1 × 1 × 1.1 mm) and a FLAIR image (1 × 1 × 1 mm) were obtained simultaneously using the integrated PET-MR system. PMOD Alzheimer’s discrimination tool (https://www.pmod.com/web/?portfolio=32-alzheimers-discrimination-for-fdg-palz) was used for image analysis. The AD t-sum score was calculated using this software as the global indices of AD-related hypometabolism^39^. We further used individual FDG-PET scans to perform spatial normalization, and then the global mean intensity in the gray matter mask was used as the reference to calculate the standard uptake value ratio (SUVr) in each region according to the AAL template^40^. The mean SUVr values from both hemispheres in AD-related regions including the middle frontal gyrus, angular gyrus, calcarine gyrus, middle temporal gyrus and posterior cingulum were calculated for further analysis^41^.

### Statistical analysis

All statistical analyses were performed using SAS version 9.2 (SAS Institute, Cary, NC, USA). Continuous and dichotomous variables are expressed as mean and standard deviation and number and percentage, respectively. Ordinal variables are presented as median and interquartile range. Socio-demographic variables included age, gender, and years of education, and the results of the neuropsychological tests including the MMSE, CDR, CDR-SB, and ECog were compared between the normally aging and early stage of probable AD groups using the chi-square test and Mann–Whitney U test. Nonparametric Spearman pairwise correlation analysis was used to study associations between the total ECog scores, total CERAD-NAB scores and their subtest scores in both groups. The raw CERAD-NAB score may have been confounded by age, gender, and years of education. Therefore, we used regression analysis to adjust for any confounding effects. The raw total CERAD-NAB scores in the normally aging group were entered into the regression analysis model using age, years of education, and gender as independent variables to generate the regression coefficients of the total equation. The variables with significant regression coefficients were used to generate the adjusted CERAD-NAB score in the whole sample. The raw total CERAD-NAB scores of all participants were converted into standardized z-scores according to the means and standard deviations of the adjusted CERAD-NAB score of the normally aging group. Receiver operating characteristic (ROC) curves were generated to investigate the accuracy of the average total ECog scores and z-transformed total CERAD-NAB scores for all participants. Moreover, areas under the curve (AUCs) with 95% confidence intervals (CIs) were calculated for each test. To compare the discriminative abilities of these tests in both groups, the AUCs were assessed using the Wald test. In addition, the cutoff points were determined according to the sensitivity and specificity. To study the association between the AD t-sum scores, regional SUVr and total and domain-specific scores of the ECog scale and z-transformed total CERAD-NAB scores, we used Spearman pairwise correlation analysis. The level of significance was set at *P* < 0.01.
